# Effects of Sex and Age on Fat Fraction, Diffusion-Weighted Image Signal Intensity and Apparent Diffusion Coefficient in the Bone Marrow of Asymptomatic Individuals: A Cross-Sectional Whole-Body MRI Study

**DOI:** 10.3390/diagnostics11050913

**Published:** 2021-05-20

**Authors:** Alberto Colombo, Luca Bombelli, Paul E. Summers, Giulia Saia, Fabio Zugni, Giulia Marvaso, Robert Grimm, Barbara A. Jereczek-Fossa, Anwar R. Padhani, Giuseppe Petralia

**Affiliations:** 1 Division of Radiology, IEO European Institute of Oncology IRCCS, 20141 Milan, Italy; luca.bombelli@ieo.it (L.B.); paul.summers@ieo.it (P.E.S.); giulia.saia@ieo.it (G.S.); fabio.zugni@ieo.it (F.Z.); 2 Division of Radiotherapy, IEO European Institute of Oncology IRCCS, 20141 Milan, Italy; giulia.marvaso@ieo.it (G.M.); barbara.jereczek@ieo.it (B.A.J.-F.); 3 Department of Oncology and Hemato-Oncology, University of Milan, 20122 Milan, Italy; giuseppe.petralia@ieo.it; 4 MR Applications Pre-Development, Siemens Healthcare, 91052 Erlangen, Germany; robertgrimm@siemens-healthineers.com; 5 Paul Strickland Scanner Centre, Mount Vernon Cancer Centre, Northwood HA6 2RN, UK; anwar.padhani@stricklandscanner.org.uk; 6 Precision Imaging and Research Unit, Department of Medical Imaging and Radiation Sciences, IEO European Institute of Oncology IRCCS, 20141 Milan, Italy

**Keywords:** bone marrow, sex and age, whole-body MRI, DWI, ADC, fat fraction, asymptomatic individuals

## Abstract

We aimed to describe the relationships between the relative fat fraction (%FF), muscle-normalized diffusion-weighted (DW) image signal intensity and water apparent diffusion coefficient (ADC), sex and age for normal bone marrow, in the normal population. Our retrospective cohort consisted of 100 asymptomatic individuals, equally divided by sex and 10-year age groups, who underwent whole-body MRI at 1.5 T for early cancer detection. Semi-automated segmentation of global bone marrow volume was performed using the DW images and the resulting segmentation masks were projected onto the ADC and %FF maps for extraction of parameter values. Differences in the parameter values between sexes at age ranges were assessed using the Mann–Whitney and Kruskal–Wallis tests. The Spearman correlation coefficient r was used to assess the relationship of each imaging parameter with age, and of %FF with ADC and normalized DW signal intensity values. The average %FF of normal bone marrow was 65.6 ± 7.2%, while nSI_b50_, nSI_b900_ and ADC were 1.7 ± 0.5, 3.2 ± 0.9 and 422 ± 67 μm^2^/s, respectively. The bone marrow %FF values increased with age in both sexes (r = 0.63 and r = 0.64, respectively, *p* < 0.001). Values of nSI_b50_ and nSI_b900_ were higher in younger women compared to men of the same age groups (*p* < 0.017), but this difference decreased with age. In our cohort of asymptomatic individuals, the values of bone marrow relative %FF, normalized DW image signal intensity and ADC indicate higher cellularity in premenopausal women, with increasing bone marrow fat with aging in both sexes.

## 1. Introduction

Whole-body MRI (WB-MRI) has become an established tool for the management of several cancer histotypes, with applications in assessing the extent of disease and evaluating response to therapy [1,2,3,4]. Its use has been extended to subjects with cancer predisposition syndromes and, thanks to the absence of ionizing radiations and the acceptable scan time, interest is growing in applying this technique for early cancer detection in the general population [5,6].

WB-MRI is of particular interest for the evaluation of bone marrow primary and metastatic lesions, where traditional imaging modalities (bone scan (BS) and computed tomography (CT)) have known limitations. On the contrary, with WB-MRI normal bone marrow and lesions can be characterized in terms of adiposity, water content and cellularity and changes in their composition can be depicted [7,8]. Furthermore, quantitative analysis of WB-MRI provides clinically relevant objective measures that can support image interpretation [9]. Relative fat fraction (%FF) obtained from T1 Dixon images, can describe bone marrow substitution occurring in bone lesions, and the apparent diffusion coefficient (ADC) calculated from different b-value diffusion-weighted (DW) functional images, can describe water diffusivity and is useful for classifying findings and response to therapy [10,11]. Functional and quantitative analysis of WB-MRI play a central role in the assessment of metastasis and multiple myeloma in METastasis Reporting and Data System for Prostate Cancer (MET-RADS) and Myeloma Response Assessment and Diagnosis System (MY-RADS) guidelines, that provide a consistent interpretation when evaluating diseased and healthy bone marrow [12,13,14]. Both guidelines point to the challenges of normal bone marrow evaluations when there is coexistent disease (active or inactive), therapy effects, myelosuppression related to therapies and bone-marrow rebound.

Apart from the changes induced by disease, normal bone marrow undergoes physiological transformations, that are common to both the general population and cancer patients [11]. It is known that bone composition differs between the sexes and that ageing thins the trabecular structure of bone, shifts the balance of bone marrow water-fat composition towards increased fat content and reduces perfusion [15,16]. Therefore, determining the normal bone marrow values and understanding the effects of physiological factors—such as sex and age—on MRI-derived quantitative and functional parameters is important in order to improve the reliability of distinction of the normal bone marrow from diseased, and enable more accurate response assessments in cancer patients.

Values of %FF, DW image signal and ADC in normal bone marrow of healthy volunteers have been reported previously, but the heterogeneity in these results is considerable across studies due to differences in hardware, acquisition settings and processing [17,18,19] that limit comparisons. Indeed, most of previous studies used dedicated acquisition protocols (e.g., lumbar vertebrae, pelvis) instead of whole-body protocols [20,21,22]. Fat fraction was computed from MR spectroscopy, 2-point T1-weighted Dixon and multi-point Dixon MRI sequences [23,24,25,26]. Differences in DW image acquisition protocols affecting ADC quantification were related to fat saturation technique—absent, based on inversion recovery or spectral—and in the set of acquired b-values—acquisition of b0 and number of b-values [26,27]. In response to these needs, MET-RADS and MY-RADS guidelines propose WB-MRI protocols with a choice of sequences, imaging parameters and post-processing optimized for radiological interpretation, that should also allow more consistency in quantitative analyses of ADC and %FF.

In this study, a WB-MRI imaging protocol that had been adapted with minor modifications from the protocols suggested by MET-RADS and MY-RADS guidelines, was used in a population of asymptomatic non-cancer individuals. This enables valid comparisons between WB-MRI acquired in the general population and cancer patients.

In this study we measure and establish values of normal bone marrow %FF values, DW images signal intensities and ADC values in a non-cancer population, and to examine their dependence on sex and age.

## 2. Materials and Methods

### 2.1. Population

The responsible Institutional Review Board approved this retrospective observational study and waived the need for specific informed consent. All individuals had provided written informed consent for the WB-MRI examination, as well as for the use of their clinical and medical information for research and scientific dissemination purposes. The participants were asymptomatic individuals who underwent WB-MRI examinations for early cancer detection as part of health screening. We excluded those individuals in whom oncological disease was found during the WB-MRI examination. If a subject had undergone more than one WB-MRI examination during the observation period, only the first examination was considered. Starting from 1 January 2017 we consecutively selected men and women to form a cohort with a uniform age distribution across age bands of ten years (30–39, 40–49, 50–59, 60–69 and ≥70). Because bone segmentation was incomplete in some cases, additional individuals were included as needed to ensure 10 subjects with a full skeleton segmentation for each 10-year age groups.

### 2.2. Acquisition Protocol

The WB-MRI examinations were performed using a 1.5 Tesla MR scanner (MAGNETOM Avanto^fit^, Siemens Healthcare, Erlangen, Germany), following the protocol summarized in Table 1 [5]. The acquisition protocol used in this study is similar to that recommended in the MET-RADS and MY-RADS guidelines [12,13]. Supplementary Table S1 summarizes the main components of the three protocols. MR signal acquisition made use of a dedicated 20-channel head/neck coil for the head/neck station, and two anterior 18-channel coils and a 32-channel posterior array coil for the body stations. The scanning protocol consisted of whole-spine sagittal fat-suppressed (STIR) T2-weighted images, along with whole-body axial T1-weighted Dixon, T2-weighted HASTE and DW images extending from the upper limit of the orbits to mid-thighs, along with sequences for regional assessments dedicated to brain and lungs. The DW acquisition consisted of four contiguous stations of fifty 5 mm thick slices acquired in free-breathing using a single-shot spin-echo echo-planar imaging sequence. Slice-wise shimming was used for the DW scans, using a prototype acquisition software provided by the machine vendor [28]. Two b-values (50 and 900 s/mm^2^) were acquired and used to compute an ADC map with mono-exponential fitting. The T1-weighted Dixon scans consisted of four contiguous packages of seventy-two 3.5 mm thick slices, each acquired in breath-hold using a dual-echo gradient echo sequence allowing creation of four sets of images (Fat, Water, In-phase and Opposed-phase) with a single sequence.

### 2.3. Image Processing and Analysis

DW and T1-weighted images were exported in DICOM format to a post-processing workstation. Processing was limited to the three lower-body stations due to a substantially stronger DW image signal intensity in the head/neck station associated with coil sensitivity. Bone marrow segmentation was performed using a semi-automated prototype software (MR Total Tumor Load, Siemens Healthcare, Erlangen, Germany), that combined automated pre-processing and computation of a volumetric ADC map (units µm^2^/s) from the b-value images using a least-squares mono-exponential fitting. This approach, previously described for the segmentation of the visible tumor load in patients with bone metastases [29,30,31], was adapted to extract as much of the normal axial skeleton marrow signal as possible. An operator selected signal intensity thresholds that were interactively applied to a simulated high b-value image stack, followed by manual editing to obtain bone marrow masks. The bone marrow masks and the ADC maps were then saved as DICOM images. The muscle signal intensities on the DW images were obtained for each subject [32], using volumetric manual segmentations of the left psoas muscle (MEVIS draw, Fraunhofer MEVIS, Bremen, Germany).

**Figure 1:**
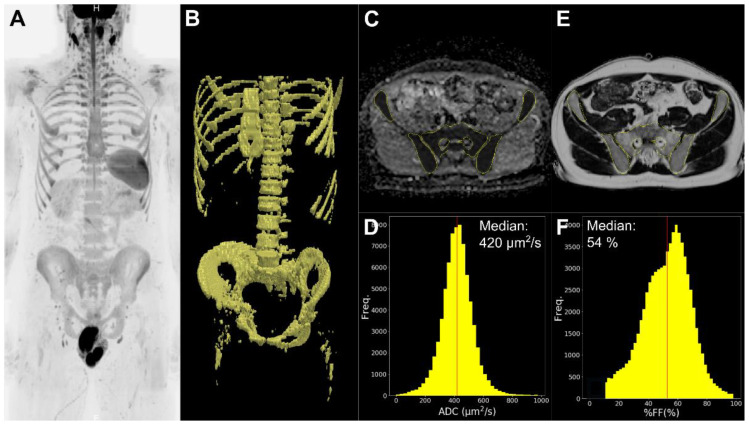
Illustration of the process for apparent diffusion coefficient (ADC) and relative fat fractions (%FF) extraction in a 30-year-old man who performed WB-MRI for early cancer detection. Based on the acquired diffusion-weighted image (b900) seen in coronal MIP with inverted grayscale (**A**), the bone marrow mask (**B**) was obtained using a semi-automated software that combines calculation of a simulated diffusion-weighted image, signal intensity thresholding, and manual editing. Only the three lower body stations were considered for the analysis because the head/neck coil array yielded different signal-to-noise levels for the head/neck station that would have necessitated a separate threshold value for semi-automated segmentation. The bone marrow mask was projected onto the ADC map computed from the diffusion-weighted image (**C**), the ADC distribution in bone marrow obtained (yellow histogram) and the median value (red line) calculated (**D**). The %FF map computed from the Dixon images was slice matched and resampled to the resolution of the diffusion-weighted images and the bone marrow mask projected onto the resampled fat fraction map (**E**) to extract the distribution of bone marrow %FF values and to calculate the median value (red line) (**F**). Voxels with %FF values below 15% or ADC above 1000 µm^2^/s were excluded from the masks to limit measure biases produced by soft tissues included in the bone marrow segmentation due to subject motion between sequence acquisitions.

Further processing was performed using ad hoc functions (Python 3.7, Python Software Foundation, Beaverton, OR, USA). First, the T1-weighted Fat and Water images were used to compute a relative %FF map (%FF = Fat/(Water + Fat) × 100). The %FF maps were aligned to the DW images using the spatial information contained in the DICOM headers, matching slices and resampling the voxels in the axial plane. Second, a muscle-normalized DW image signal intensity was calculated for each voxel in the b50 (nSI_b50_) and b900 (nSI_b900_) DW images by dividing the signal values by the median muscle signal intensities at the same b-value. Third, bone marrow masks were refined by excluding voxels with ADC values above 1000 μm^2^/s (i.e., above the range of normal bone marrow ADC values [32]) or with %FF values below 15% (the minimum values of fat fraction in bone, reported in the literature [23,26]) in order to limit measure biases produced by residual soft tissues included in the bone marrow segmentation and image noise. Finally, the bone marrow median %FF, nSI_b50_, nSI_b900_ and ADC values for each subject were computed by projecting the refined bone marrow mask onto the respective images (Figure 1).

### 2.4. Statistical Analysis

The mean values, lower and upper limits for each parameter, defined as mean ± 2SD for parameters with a normal distribution and 5th and 95th percentiles for non-normal ones (Shapiro–Wilk test) were reported for the entire cohort. An ANCOVA test was performed to investigate the effect of sex and age on relative %FF values, nSI_b50_, nSI_b900_ and ADC values, and significant variables were included in linear models to predict %FF and ADC values. Further analyses were performed on subgroups formed by dividing the population by sex and 10-years age groups. A Mann–Whitney U test was used to compare the values between sexes, and Kruskal–Wallis test to compare parameter values across the age-bands for each sex. Finally, the correlations of ADC, nSI_b50_ and nSI_b900_, with age and %FF were assessed using the Spearman coefficient (r). Results with *p*-values < 0.05 were considered significant. The statistical analysis was performed using the R software (R 3.5.1, R Foundation for Statistical Computing, Vienna, Austria).

## 3. Results

### 3.1. Population

The main analysis was based on 100 examinations of individuals equally divided by sex and age: the 50 men were aged between 30 and 81 years (average age 54.7 years), with an average body mass index (BMI) of 25.6 ± 3.3 kg/m^2^, and the 50 women were aged between 30 and 79 years (average age 54.7 years), with an average BMI of 23.3 ± 3.3 kg/m^2^ (Table 2).

**Figure 2:**
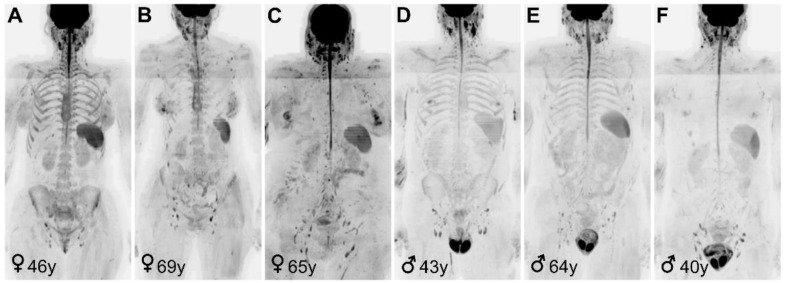
High b-value (b = 900 s/mm^2^) inverted gray-scale coronal maximum-intensity-projection images of 6 asymptomatic individuals. (**A**) In our cohort, the bone marrow signal intensity of women (♀) aged between 30 and 50 was higher compared to the bone marrow signal of older women (**B**) and adult men (♂) of all age groups (**D**,**E**). This difference may be attributable to the higher cellularity, water content and possibly higher perfusion of pre-menopausal women bone marrow. Images (**C**,**F**) show two examples of individuals for which the semi-automatic segmentation method used in this study was not applicable, due to hypointense bone marrow signal intensity on high b-value inverted gray-scale coronal maximum-intensity-projection images.

Due to low bone marrow signal in the DW images, it was necessary to replace 12 subjects by an equal number of individuals of the same sex and age group, for whom the semi-automated segmentation of bone marrow succeeded. The 12 examinations where semi-automated segmentation failed were subject of a separate *post hoc* analysis investigating differences with the examinations where the segmentation succeeded (Appendix A). The heterogeneity of bone marrow appearance on DW images is shown in Figure 2.

### 3.2. Overall Distribution of Relative %FF, Signal Intensity in DW Images and ADC and Their Correlation with Age

Considering the 100 segmented WB-MRI examinations, the average (±standard deviation) relative %FF of bone marrow was 65.6 ± 7.2%, while the average nSI_b50_, nSI_b900_ and ADC of bone marrow were 1.7 ± 0.5, 3.2 ± 0.9 and 422 ± 67 μm^2^/s, respectively. The lower and upper limit values of %FF, nSI_b50_, nSI_b900_ and ADC of normal bone marrow are summarized in Table 3.

There was a moderate positive correlation between age and %FF, with r = 0.63 (*p* < 0.001). On the contrary, there was a weak negative correlation between age and the parameters derived from the DW images, with Spearman coefficients r of −0.30, −0.27 and −0.23 (highest *p* = 0.02), respectively, for nSI_b50_, nSI_b900_ and ADC (Table 4). The correlations of ADC, nSI_b50_ and nSI_b900_ are reported in Appendix A.

### 3.3. Effect of Sex and Age on Relative %FF and Signal Intensity in DW Images and ADC

Age was the only significant predictor of relative %FF (*p* < 0.001). A simple regression model was calculated to predict %FF based on age (F(1,98) = 61.84, *p* < 0.001, Adjusted R^2^ = 0.38). Equation (1) expresses the age dependence of %FF (%) in our cohort of normal individuals aged between 30 and 80 years of age:

(1) %FF=Age×0.3+49,

where Age is expressed in years. This means that the yearly increase of %FF in our cohort was 0.3%. For example, according to the equation, the average %FF value for a 30-year-old individual (man or woman) will be 30×0.3+49=58%, while for an 80-year-old individual it will be 80×0.3+49=73%. Both sex and age were significant predictors (*p* < 0.001 and *p* = 0.005, respectively) of nSI_b50_, nSI_b900_ and of ADC. The interaction term between sex and age was significant for nSI_b50_ and nSI_b900_, (*p* = 0.003), with the difference in values between men and women decreasing with age, however it was not significant for ADC (*p* = 0.065). Equation (2) expresses the multiple linear regression model that considers sex and age (F(2,97) = 29.41, *p* < 0.001, Adjusted R^2^ = 0.36), for the determination of ADC value (µm^2^/s) in our cohort:

(2) ADC=480+Sex×53−Age,

where Sex equals 0 for men and 1 for women and Age is expressed in years. This means that the ADC value was 53 µm^2^/s higher for women and that it decreased by 1 µm^2^/s every year. Equation (2) can be split by sex to obtain the simpler expressions (Equations (3) and (4)):

(3) $${\mathrm{ADC}}_{\mathrm{men}}=480-\mathrm{Age}\;\mathrm{and}$$

(4) $${\mathrm{ADC}}_{\mathrm{women}}=533-\mathrm{Age}.$$

After performing ANCOVA, we separately analyzed the effects of sex and age on the considered WB-MRI parameters. Analysis of the men and women sub-populations (Table 3) showed that average %FF values were similar between the sexes (*p* = 0.767), while average values of nSI_b50_, nSI_b900_ and ADC were significantly higher in women than in men (*p* = 0.049, *p* < 0.001 and *p* < 0.001, respectively). The bone marrow values of nSI_b50_ were between 1.0 and 2.6 in women and 1.1 and 2.3 in men, while the values of nSI_b900_ were between 2.0 and 4.9 in women and between 2.0 and 4.0 in men. The normal bone marrow ADC values were between 345 and 532 μm^2^/s in women and between 323 and 449 μm^2^/s in men (Table 3).

The %FF values increased with age both for women and men. Indeed, we observed significant positive correlations (r = 0.63 and r = 0.64, respectively, *p* < 0.001). On the contrary, nSI_b50_, nSI_b900_ and ADC values decreased with age in women, but not in men. In women there was a significant negative correlation between age and both nSI_b50_ (r = −0.54 (*p* < 0.001)) and nSI_b900_ (r = −0.57 (*p* < 0.001)), as well as for ADC (r = −0.38 (*p* = 0.007)), but in men no significant correlations were observed (r = −0.01 (*p* = 0.949), r = 0.01 (*p* = 0.955) and r = −0.17 (*p* = 0.250), respectively). Correlation coefficients of %FF, nSI_b50_, nSI_b900_ and ADC with age for men and women are reported in Table 4.

Comparing the parameter values measured in men and women dividing the population in 10-years age groups (30–39, 40–49, 50–59, 60–69 and ≥70 years old), we observed that values of %FF were significantly different between the age groups, both for men and women (*p* < 0.001 and *p* < 0.001, respectively). Bone marrow nSI_b50_, nSI_b900_ and ADC values were significantly different between age groups in women (*p* < 0.003 for each parameter), but no differences were observed in men (*p* > 0.744 for each parameter).

**Figure 3:**
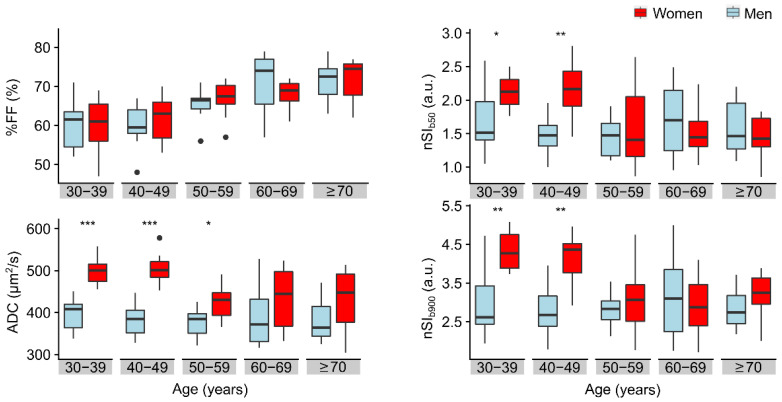
Comparison of the distributions of %FF, ADC, nSI_b50_ and nSI_b900_ values measured in bone marrow, by sex and age groups. It is clear that fat fraction (%FF) values increase with age for both men and women. The ADC, nSI_b50_ and nSI_b900_ values are higher in women between 30 and 49 years than in men of corresponding age groups (asterisks denote the significance level: * *p* < 0.05, ** *p* < 0.01, *** *p* < 0.001). ADC, nSI_b50_ and nSI_b900_ values in women were lower in the three subsequent age groups (50 years and older), probably due to menopause-induced changes in bone marrow composition, but a significant difference was observed in the ADC values of women and men of the 50–59 age group. On the contrary, the values of ADC, nSI_b50_ and nSI_b900_ measured in men were roughly constant across age groups.

Comparing women and men in the separate age groups, %FF values of men and women were not significantly different in any of the age groups (*p* = 0.256). On the contrary, the values of nSI_b50_ and nSI_b900_ were significantly different between men and women in the 30–39 years (*p* = 0.017 and *p* = 0.003, respectively) and the 40–49 years (*p* = 0.003 and *p* = 0.001, respectively) age groups. Finally, the sex difference in the ADC values was significant for the three younger groups (*p* < 0.001, *p* < 0.001, and *p* = 0.017, respectively), but not for the 60–69 and ≥70 age groups (Figure 3).

## 4. Discussion

We evaluated normal bone marrow values of quantitative WB-MRI parameters in a non-cancer adult population for their dependence on sex and age, using a clinical acquisition protocol similar to the protocols recommended for diagnosis of primary and secondary bone marrow lesions.

In our cohort, the global bone marrow relative %FF values ranged between 51% and 80% in the overall population with values increasing with age by approximately 3% per decade in both sexes. The bone marrow signal intensities (nSI_b50_ and nSI_b900_) and ADC were inversely correlated with age in women, but not in men, with higher values found in women aged 30–50 compared to older women and men of all age groups. The overall average ADC of normal bone marrow was 422 ± 67 μm^2^/s. With ADC values being higher in women than in men, the upper limit of the normal bone marrow ADC was 449 μm^2^/s in men, and 532 μm^2^/s in women.

To appreciate the appearance of bone on clinical MRI images it is important to consider its structural composition. Cortical bone, a dense hard tissue that forms the walls of all bones, contains a very low quantity of mobile water and does not generate a visible signal at the echo times used in this study [33]. Trabecular bone, on the other hand, consist not only of the hard-bony trabeculae that do not provide signal, but also of fat and hematopoietic cells, extracellular water and blood vessels that fill the porous matrix formed by the trabeculae. These soft components constitute the bone marrow and contribute to the signal in a typical WB-MRI examination. Bone marrow is referred to as “red” marrow when it is rich in blood-forming cells and “yellow” when is composed mainly of adipocytes. It is known that a conversion of red hematopoietic marrow to yellow fatty marrow occurs with ageing. This transformation begins at birth, when all bones contain haematopoietically active marrow. At the age of 25 red marrow is limited to axial skeleton, ribs and breastbone, and the yellow marrow conversion continuing with ageing [15].

We observed that the global values of relative %FF from whole-body bone marrow were between 50% and 80%. In previous studies that used MR spectroscopy [23,34] and proton density fat fraction [24] for bone marrow adiposity quantification in lumbar vertebrae, the reported fat fraction values were low compared to ours, ranging between 15% and 60%. The differences may be attributed to our inclusion of skeletal regions with higher fat content compared to lumbar vertebrae (e.g., pelvis), as it has been shown that a large heterogeneity in bone marrow fat fractions exists across anatomical regions [35]. In addition, the 2-point T1-weighted Dixon images used for calculating the relative %FF in our study are sensitive to T1 weighting and possibly T2 decay effects [36], that may lead to measurement bias.

The relative %FF values increased with age by an average of 3% per decade in our cohort of adult men and women. Increasing bone marrow adiposity with age in lumbar vertebrae is consistent with previous reports [23,24,34] and likely reflects the known process of conversion of red cellular marrow to yellow fatty marrow. The sex differences in bone marrow adiposity reported in the literature are attributed to the high estrogen levels of women in reproductive age, steeply decreasing with menopause, compared to the more stable levels of adult men [23]. Indeed, a protective effect of estrogens has been shown against marrow adiposity [37,38]. These differences were not evident in our results. In particular, we observed no significant difference in relative %FF between sexes at any age. This may again be attributed to the inclusion of relatively large quantities of yellow marrow in our bone segmentation, as well as the use of T1-weighted Dixon as the fat quantification technique, as relative %FF may be less precise in bone marrow fat quantification compared to MR spectroscopy and proton density fat fraction [39].

Increasing adiposity of bone marrow is expected to be counterbalanced by a reduction of its water content [40]. Water content is reflected by our values of nSI_b50_ as, despite the mild diffusion-weighting, they are somewhat T2-weighted and are subject to a fat saturation preparation. In our cohort the average nSI_b50_ value was 1.7 ± 0.5, but younger women (those of 30–39 and 40–49 age groups) had higher values than older women, while men had lower and almost constant values across age groups. This is consistent with previous studies, where women, especially those in reproductive age, had higher bone marrow cellularity and water, and longer T2 relaxation times [41,42] compared to men. Furthermore, the literature indicates that the largest bone marrow water content decline occurs after 45 years in women but before 25 years in men, and then remains relatively stable [43].

Direct comparisons with nSI_b50_ in other studies is not possible but the normal bone marrow nSI_b900_ was subject of previous studies and, consistently with our results, higher values were found in younger women compared to older women and adult men of any age [27,32,42,44]. The higher bone marrow nSI_b900_ may be predominantly attributed to the T2 signal shine-through in the diffusion-weighted signal when marrow water content is high in younger women.

The technique used for segmentation in this study depends on the signal intensity difference between bone marrow and other tissues. Thanks to the high nSI_b900_ signal of bone, relative to other tissues in the younger women of our cohort, the segmentation was easily performed. On the contrary, in 12 individuals it was not possible to segment bone marrow using the signal intensity threshold. Indeed, their bone marrow was sometimes hypointense compared to soft tissues. BMI and %FF were higher in the 12 individuals, while nSI_b50_ and nSI_b900_ were lower and ADC values similar, suggesting an effect of body composition, and particularly of bone marrow fat content on signal of DW images. If BMI has an effect on %FF and ADC, the fact that the population of the main analysis has similar BMI with moderate variability (25 Kg/m^2^), might limit considerations to populations with different average BMIs. If the high nSI_b50_ is seen as a T2 shine-through effect, the low nSI_b50_ in these 12 subjects is suggestive of a shortening of transverse relaxation times. Our results, however, do not provide further insight into the mechanisms involved, though several possibilities exist. Differences in the proportions of adipocyte types in the marrow (brown versus yellow which differ in size and lipid content [15]) and adipocyte distribution patterns within the trabecular spaces (fat cells occupying predominantly the center of marrow spaces or being scattered within hematopoietic tissue [45]) may have an effect on both nSI_b50_ and nSI_b900_ values. The participants come from a population where thalassemia is relatively common, so T2 reduction due higher concentrations of iron in bone marrow is also a possibility, but their status for this trait was not assessed [46]. A further possibility relates to differences in trabecular architecture, thickness and marrow space dimensions, modulating the internal gradients associated with the magnetic susceptibility difference between bone and bone marrow. Such inhomogeneities shorten T2* relaxation and may thus reduce the marrow signal intensity on b50 and b900 images in echo-planar imaging, as well as on Dixon images with consequences for the calculation of relative %FF, but are not expected to influence ADC [17].

The average ADC of bone marrow was 422 ± 67 μm^2^/s in our cohort. The literature has focused on the evaluation of lumbar vertebrae, where ADC values ranging 200–600 μm^2^/s have been reported, which are lower than most other normal tissues, where values are typically between 700 and 2400 μm^2^/s [17]. The presence of the hydrophobic adipocytes can be expected to cause substantial diffusion tortuosity and reduce the effective diffusion length of the water molecules in bone marrow [40]. Indeed, we observed a negative correlation of ADC with relative %FF, consistent with some previous reports [26,42]. The literature on this point is mixed however, likely due to factors such as the choice of a spectral fat suppression technique for the diffusion-weighted images acquisition [20,27].

Higher ADC values were observed in women than in men, with the upper limit of the normal bone marrow ADC values being 449 μm^2^/s for men and 532 μm^2^/s for women, and younger women had higher ADC values than older women, while men had lower and almost constant values with age. A negative correlation between age and bone marrow ADC values in women has been reported previously both in WB-MRI studies [26,42,44], and in studies using regional protocols [21,47,48,49], while the correlation of bone marrow ADC values and age in men has been reported to be slight or null [42,50]. This may be attributed to the higher bone marrow water content of younger women, allowing for longer effective diffusion lengths. Perfusion and intra-medullary blood flow, which are higher in the bone marrow of young women [51], may also influence the bone marrow ADC values by contributing to the signal of the low b-value image. With the b-values used in this study, it was not possible to calculate the perfusion contribution to the ADC. A difference in the age dependence of ADC values between men and women was not evident in our regression model (the interaction term between age and sex was not significant, *p* = 0.065). The relatively high variability in the ADC values of older women likely compromised fitting of the linear model, but ADC values in women below 50 years of age were well-separated from those of both older women and men of any age.

Our results help to define the characteristics of normal bone marrow in asymptomatic subjects as seen with WB-MRI. WB-MRI being increasingly recommended for the management of patients with malignant bone marrow disease [1,2,3], the knowledge of the distributions of quantitative parameters in normal bone marrow on clinical WB-MRI acquisition protocols has implications for their management. First, our results are useful for distinction between normal bone marrow and malignant bone marrow deposits. Indeed, we found that sex and age both influence ADC values of normal bone marrow and therefore they should both be considered when setting the thresholds for distinguishing normal bone marrow from malignant bone marrow deposits in the quantitative analysis of WB-MRI in metastatic patients. For example, in younger women, the higher signal intensity of normal bone marrow on high b-value DW images and higher ADC values compared to other age and sex groups, may make differentiation of malignant bone marrow deposits more challenging. Second, our results are useful to monitor how cancer and cancer therapies affect bone marrow health. Indeed, they both promote the acceleration of bone remodeling process, inducing trabecular resorption and increasing the risks for both bone loss and fracture development [52]. As relative %FF and ADC are being investigated as markers of bone quality [18,19,53], the values measured in this study may be considered a reference for normal bone marrow and could be compared with the values measured in the normal bone marrow of cancer patients undergoing WB-MRI during therapy to assess therapy-induced bone weakening. This is possible because the acquisition protocol used in this study is comparable to the protocols recommended in MY-RADS and MET-RADS guidelines for the radiological reporting of WB-MRI in cancer patients: adoption of these recommendations supports greater standardization of quantitative assessments, such as fat fraction and ADC, by standardizing sequences used, sets of b-values acquired and post-processing.

As the primary limitation of this study, we note that several factors were not considered, that may have had an effect on relative %FF, signal of DW images and ADC measures, such as bone mineral density and trabecular architecture, blood markers (e.g., hemoglobin and sex hormones), lifestyle (smoking, physical activity, high-altitude living, etc.) and, for women, menopausal status. While no cases of vertebral collapse, Modic changes, Schmorl’s hernias or spondylodiscitis, beyond age-consistent limits, were noted by the radiologists, we have not excluded regions of focal alterations. This may be a source of variability, but is expected to have little impact on the overall ADC and %FF values as they would account for a small fraction of the segmented bone volume. Further, the imaging parameters were those optimized for radiological interpretation and not necessarily for quantification. Being consistent with the MET-RADS and MY-RADS recommendations, it should be possible to closely replicate the protocol on other MR scanners in order to test generalizability of our results. However, DW image signal intensities can be lower on 3T scanners due to trabecular bone causing T2* effects. It is recognized that ADC values are influenced by acquisition settings such as fat saturation and selection of b-values. This has contributed to a wide variability in ADC values in the literature. Implementations of measures to correct for position dependent DW imperfections is an open area of research that could help reduce ADC variability [54]. Similarly, relative %FF values are sensitive to T2* effects, which are not compensated for in the dual echo Dixon calculation, as well as to T1-weighting, potentially biasing the marrow adiposity quantification. For dedicated studies of fat fraction the use of proton density fat fraction measurements is recommended [55], but in the context of clinical studies the extra-time required needs to be balanced against the priorities of radiological assessment. While our values for ADC and relative %FF are in the range of published values, the specific trends observed may be influenced by the limitations of the underlying measurements. Moreover, while the sample was large enough to identify global trends and values, a larger number of subjects per age group would be needed in order to obtain robust characterization of sex and age-adjusted cut-off criteria for normal bone marrow. Similarly, all image acquisitions were performed on the same 1.5T MR scanner and generalizability should be assessed using different acquisition systems. Finally, the segmentations were evaluated only as a whole, without distinguishing between red and yellow bone marrow and without distinguishing between different skeletal regions, which are known to present differences. This region-specific analysis could be the subject of a future study.

## 5. Conclusions

In conclusion, women aged between 30 and 50 years had higher ADC values and signal on DW images, compared to older women and men of any age. In addition, ADC values and signal on DW images were negatively related to age in women, a trend that was not observed in men. Relative %FF values increased with age for both men and women without differences between sexes. Together, these observations manifested in a negative correlation between ADC and relative %FF that reflects the important role of both the fat and water content of bone marrow in determining the diffusion imaging signals.

## Figures and Tables

**Table 1:** Whole-body MRI sequence parameters of the protocol used for early cancer detection.

Parameter	DWI	T1W	T2W
Sequence type	SS-EPI	GRE Dixon	HASTE
FOV (mm)	430	430	430
Phase FOV (mm)	390.9	364.0	335.9
Matrix (phase × freq.)	120 × 132	209 × 352	175 × 320
Voxel (mm)	1.6 × 1.6 × 5	1.2 × 1.2 × 3.5	1.3 × 1.3 × 5
*n* of slices/station	50	72	16
*n* of stations	4	4	12/13
Slice thickness (mm)	5	3.5	5
Gap between slices (mm)	0	0.7	1
TR (ms)	6550	6.65	800
TE (ms)	62	2.39/4.77	74
TI (ms)	180	-	-
Flip angle (degrees)	90	20.5	149
*n* of averages	5 (b50)/15 (b900)	1	1
Fat suppression	STIR	-	-
b-values (s/mm^2^)	50, 900	-	-
Breathing	Free	Hold	Hold
Acquisition time per station (min:s)	3:46	0:16	0:16
Image plane	Transversal	Transversal	Transversal

Notes: DWI = Diffusion-Weighted Images, FOV = Field of View, GRE = Gradient Echo, HASTE = Half-Fourier Acquisition Single-Shot Turbo Spin Echo, SS-EPI = single-shot spin-echo echo-planar imaging, STIR = Short-Tau Inversion Recovery, T1W = T1 Weighted, T2W = T2 Weighted, TE = Echo Time, TI = Inversion Time, TR = Repetition Time.

**Table 2:** Characteristics of individuals included in the study.

Population	Age Range (Years)	Men		Women	
		*n*	BMI (kg/m^2^)	*n*	BMI (kg/m^2^)
Overall	30–81	50	25.6 (3.3)	50	23.3 (3.3)
10-years age groups	30–39	10	26.7 (4.6)	10	23.5 (3.8)
	40–49	10	24.9 (2.6)	10	22.7 (3.0)
	50–59	10	25.0 (2.7)	10	24.7 (2.9)
	60–69	10	25.5 (3.1)	10	23.0 (3.6)
	≥70	10	25.7 (3.3)	10	22.7 (3.1)

Notes: Values are mean (standard deviation), BMI = Body Mass Index.

**Table 3:** Comparisons of imaging-derived apparent diffusion coefficient, normalized diffusion weighted signal intensity and relative fat fraction values for bone marrow.

Parameter	Value Descriptors	Full Cohort (N = 100)	Men (N = 50)	Women (N = 50)	*p*-Value
%FF (%)	Mean (SD)	65.6 (7.2)	65.5 (7.7)	65.7 (6.6)	0.767
	±2SD	51.2–80.0	50.1–80.9	52.5–78.9	
nSI_b50_ (a.u.)	Mean (SD)	1.7 (0.5)	1.6 (0.4)	1.8 (0.5)	0.049
	5th–95th percentile	1.0–2.5	1.1–2.3	1.0–2.6	
nSI_b900_ (a.u.)	Mean (SD)	3.2 (0.9)	2.9 (0.7)	3.5 (0.9)	<0.001
	5th–95th percentile	2.0–4.8	2.0–4.0	2.0–4.9	
ADC (µm^2^/s)	Mean (SD)	422 (67)	384 (46)	460 (63)	<0.001
	5th–95th percentile	328–524	323–449	345–532	

Note: Values are mean (standard deviation). *p*-values from Mann–Whitney U test for significance of men vs. Women difference. ADC = apparent diffusion coefficient, nSI_b50_ = muscle-normalized b50 signal intensity, nSI_b900_ = muscle-normalized b900 signal intensity, %FF = relative fat fraction. 2SD and 5th–95th percentiles were used to indicate the value limits for normally and non-normally distributed parameters (Shapiro–Wilk test), respectively.

**Table 4:** Correlation with age of imaging-derived apparent diffusion coefficient, normalized diffusion weighted signal intensity and relative fat fraction values for bone marrow.

Parameter	Value Descriptors	Full Cohort (N = 100)	Men (N = 50)	Women (N = 50)
%FF (%)	r	0.63	0.63	0.64
	*p*-value	<0.001	<0.001	<0.001
nSI_b50_ (a.u.)	r	−0.3	−0.01	−0.54
	*p*-value	0.003	0.949	<0.001
nSI_b900_ (a.u.)	r	−0.27	0.01	−0.57
	*p*-value	0.006	0.955	<0.001
ADC (µm^2^/s)	r	−0.23	−0.17	−0.38
	*p*-value	0.02	0.250	0.007

Note: ADC = apparent diffusion coefficient, nSI_b50_ = muscle-normalized b50 signal intensity, nSI_b900_ = muscle-normalized b900 signal intensity, r = Spearman correlation coefficient, %FF = relative fat fraction.

**Figure A1:**
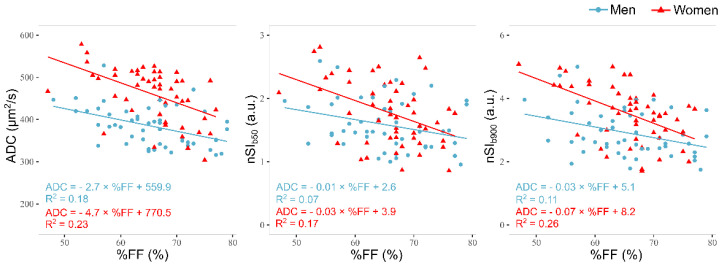
Scatterplots showing the correlation of apparent diffusion coefficient (ADC, left), muscle-normalized signal intensity of b50 and b900 images (nSI_b50_, middle, and nSI_b900_, right) and the fat fraction values (%FF). Linear regression lines and equations are reported.

## Data Availability

The data is available for review from the corresponding author on request.

## Supplementary Materials

The following are available online at https://www.mdpi.com/article/10.3390/diagnostics11050913/s1, Table S1. Sequence components for the WB-MRI acquisition protocol used in this study compared to MY-RADS and MET-RADS protocols.

## Appendix A

### Appendix A.1. Correlation of Relative %FF with Signal Intensity in DW-Images and ADC

The relative %FF values were negatively correlated with nSI_b50_, nSI_b900_ and ADC (Figure A1). The correlations were significant in women (r = −0.39 for nSI_b50_, r = −0.53 for nSI_b900_ and r = −0.55 for ADC, highest *p* = 0.007). The correlations were weaker in men, remaining significant for nSI_b900_ and ADC (r = −0.31, *p* = 0.031 and r = −0.43, *p* = 0.002, respectively), while for nSI_b50_ there was a non-significant negative trend (r = −0.28, *p* = 0.053).

### Appendix A.2. Post Hoc Analysis

The semi-automated segmentation failed on DW-images of 10 men aged between 30 and 70 years (mean age 47.3 years), with an average BMI of 27.2±4.2 kg/m^2^, and 2 women aged 44 and 65 years, with BMIs of 24.7 and 31.2 kg/m^2^. The BMI values of these 12 individuals were significantly higher than in the population of the main analysis (*p* = 0.032). In these 12 individuals we performed manual segmentation of lumbar and pelvic vertebrae on the b50 images. The subsequent processing steps were the same as used for above analysis based on semi-automated segmentation.

The average bone marrow relative %FF measured in the 12 individuals was 71.1 ± 7.2% and was significantly higher (*p* = 0.014, Mann-Whitney U test) compared to the values measured in the population of the main analysis. On the contrary, nSI_b50_ and nSI_b900_ values, respectively 0.7 ± 0.2 and 1.2 ± 0.2, were significantly lower (both *p* < 0.001). The ADC values were 414 ± 32 μm^2^/s and were not significantly different from those found in the population of the main analysis (*p* = 0.692).

As our results highlighted sex differences, a further comparison was made between the values measured in the 50 men of the main analysis and those measured in the 10 men segmented manually. Both nSI_b50_ and nSI_b900_ were significantly lower (*p* < 0.001) in the group of the manual segmentations, but no significant differences were found for ADC (*p* = 0.146) or %FF (*p* = 0.058).

In order to understand whether acquisition problems may have reduced the signal intensity in the whole acquired volume, we also compared the psoas muscle signal intensities between the two groups, but no significant differences were found for either b50 (*p* = 0.393) or b900 (*p* = 0.129) images. These comparisons were not repeated for women as only two were segmented manually.
