# Supplementary material for: Effects of Sex and Age on Fat Fraction, Diffusion-Weighted Image Signal Intensity and Apparent Diffusion Coefficient in the Bone Marrow of Asymptomatic Individuals: A Cross-Sectional Whole-Body MRI Study
Source: Diagnostics (Basel). 2021 May 20;11(5):913. doi: 10.3390/diagnostics11050913 (PMC8161193; doi:10.3390/diagnostics11050913)
Supplement: Supplementary file 1 [file diagnostics-11-00913-s001.zip › diagnostics-1221075-supplementary.pdf]

Article

Effects of Sex and Age on Fat Fraction, Diffusion-Weighted Image Signal Intensity and Apparent Diffusion Coefficient in the Bone Marrow of Asymptomatic Individuals: A Cross-Sectional Whole-Body MRI Study

Supplementary Table S1. Sequence components for the WB-MRI acquisition protocol used in this study compared to MY-RADS and MET-RADS protocols.

Sequence description	MY-RADS [1]	MET-RADS [2]	Our study
Whole spine – sagittal, T1W TSE, 4–5 mm SLT	Yes	Yes	No
Whole spine – sagittal T2W TSE STIR (preferred) or fat suppressed, 4–5 mm SLT	Yes	Yes	Yes
Whole-body – axial, T1W GRE Dixon, 5 mm SLT<br>Fat image reconstructions mandatory	Yes (vertex to knees)	Yes (vertex to mid-thighs)	Yes (orbits to mid-thighs)
Whole-body – axial, diffusion-weighted STIR, 5–7 mm SLT<br>- b50-100 s/mm² and b800-1000 s/mm²<br>- ADC calculations with mono-exponential data fitting<br>- Coronal b800–1000 multiplanar reconstruction<br>- 3D-MIP reconstructions of highest b-value images	Yes (vertex to knees)	Yes (skull base to mid-thighs)	Yes (orbits to mid-thighs)
Whole-body – axial, T2W TSE without fat-suppression, 5 mm SLT - preferably matching the diffusion-weighted images	Optional (vertex to knees)	Optional (vertex to mid-thighs)	Yes (orbits to mid-thighs)
Regional assessments	Optional	Optional	Brain - T2W FLAIR<br>Lung - T1W GRE

Notes: ADC=apparent diffusion coefficient, FLAIR=Fluid Attenuated Inversion Recovery, GRE=Gradient Echo, MIP=maximum intensity projection, SLT=slice thickness, STIR=Short Tau Inversion Recovery, TSE=Turbo Spin Echo

References:

1. Messiou, C.; Hillengass, J.; Delorme, S.; Lecouvet, F.E.; Moulopoulos, L.; Collins, D.J.; Blackledge, M.D.; Abildgaard, N.; Østergaard, B.; Schlemmer, H.-P.; et al. Guidelines for Acquisition, Interpretation, and Reporting of Whole-Body MRI in Myeloma: Myeloma Response Assessment and Diagnosis System (MY-RADS). *Radiology* **2019**, *291*, 5–13, doi:10.1148/radiol.2019181949.
2. Padhani, A.R.; Lecouvet, F.E.; Tunariu, N.; Koh, D.-M.; De Keyzer, F.; Collins, D.J.; Sala, E.; Schlemmer, H.P.; Petralia, G.; Vargas, H.A.; et al. METastasis Reporting and Data System for Prostate Cancer: Practical Guidelines for Acquisition, Interpretation, and Reporting of Whole-body Magnetic Resonance Imaging-based Evaluations of Multiorgan Involvement in Advanced Prostate Cancer. *Eur. Urol.* **2017**, *71*, 81–92, doi:10.1016/j.eururo.2016.05.033.
